# An Uncommon Manifestation of a Great Imitator: Gummatous Syphilis of the Liver in an HIV-Positive Patient

**DOI:** 10.1155/2024/6571155

**Published:** 2024-10-21

**Authors:** Nadia Solomon, Tom Heller, Tommaso Manciulli, Paola Del Giacomo, Katleen de Gaetano Donati, Enrico Brunetti, Francesco Taccari

**Affiliations:** ^1^Department of Radiology and Biomedical Imaging, Yale School of Medicine, New Haven, USA; ^2^Lighthouse Clinic, Lilongwe, Malawi; ^3^International Training and Education Center for Health, University of Washington, Seattle, Washington, USA; ^4^Department of Experimental and Clinical Medicine, University of Florence, Florence, Italy; ^5^Safety and Bioethics Department, Sacred Heart Catholic University, Rome, Italy; ^6^Department of Clinical-Surgical, Diagnostic and Pediatric Sciences, University of Pavia, Pavia, Italy; ^7^Unit of Infectious Diseases, IRCCS San Matteo Hospital Foundation, Pavia, Italy

**Keywords:** gumma, hepatic abscess, sexually transmitted infection, syphilis, *Treponema pallidum*, ultrasound

## Abstract

Syphilis is a sexually transmitted infection caused by *Treponema pallidum*. It progresses in phases and undiagnosed disease can cause considerable morbidity. Tertiary syphilis causes the formation of gummas. Liver involvement is rarely described and usually limited to transaminase elevation during primary syphilis. We present a case of tertiary syphilis in an HIV patient. Microbiological, clinical, and radiological information were retrieved from the patient's record. Gummatous syphilis is rarely described in the literature, and practicing physicians should be aware of its existence and include this manifestation in the differential diagnosis of patients with a positive serology and focal liver lesions.

## 1. Background

Syphilis is a sexually transmitted infection (STI) caused by the spirochete *Treponema pallidum* and progresses in phases [1]. Although the introduction of antibiotics has led to a decline in the incidence and prevalence of syphilis, syphilis has re-emerged particularly among HIV-positive patients and men who have sex with men (MSM) [2]. Moreover, the growing use of pre-exposure prophylaxis (PrEP) for HIV and increased risk behaviour has been associated with an increased incidence of syphilis, underlining the need to strengthen appropriate monitoring programs [2, 3].

While in primary syphilis, a characteristic genital chancre may be seen, secondary syphilis, the dissemination phase, may present with a maculopapular rash often including palms and soles, lymphadenopathy, and a variety of nonspecific symptoms, including fatigue, fever, weight loss, and abdominal pain [1, 4]. During this phase, hepatomegaly reportedly occurs in approximately half of cases, with lymphadenopathy occurring in about a third and splenomegaly in just under a sixth [4]. In HIV-positive patients in particular, the potential for hepatic involvement is substantial; while studies estimate an overall prevalence of syphilitic hepatitis at 2.7%, the incidence of syphilitic hepatitis has been reported in HIV coinfection at rates ranging from 38% to 61% [4]. In the early phase of syphilis (infection duration of less than 2 years), a study by Palacios et al. reported that approximately one-third of patients coinfected with HIV demonstrated hepatic involvement, although this assertion was limited to laboratory testing and imaging findings were not assessed [4]. As syphilitic hepatitis is frequently asymptomatic—in a case series evaluating 62 patients, none of the patients demonstrated clinical signs of hepatitis [5]—the diagnosis typically mainly depends on the presence of elevated liver function tests (LFTs) and a positive treponema serology [4]. Syphilitic “granulomatous” hepatitis has also been described, presenting with a cholestatic pattern with disproportionately increased alkaline phosphatase and gamma-glutamyl transpeptidase [6]. This is consistent with liver biopsy findings, including infiltration of inflammatory cells, granuloma formation with multinucleated giant cells, cholestasis, and inflammatory bile duct infiltration [1, 4, 6].

Descriptions of imaging findings associated with syphilitic hepatitis are scarce, but one case study described the nonspecific finding of diffusely increased hepatic parenchymal echogenicity in an HIV-negative patient ultimately diagnosed with syphilitic hepatitis during the dissemination stage of the disease; in this case, no granulomatous changes were described on histopathological examination to suggest granulomatous hepatitis [7].

Here, we describe a case of gummatous hepatitis in tertiary syphilis in an HIV-positive patient with well-controlled viral load.

## 2. Case Description

A 37-year-old man presented with a two-month history of dull right upper quadrant pain, irregular fever, and night sweats. He had immigrated 15 years ago from Romania to Italy and had last traveled to Romania 1 year ago. He reported having sex with men and reported having a well-controlled HIV infection, first diagnosed 11 years prior and on therapy with emtricitabine/tenofovir alafenamide/rilpivirine (FTC/TAF/RPV). At the time of HIV diagnosis, he had also been diagnosed with late-latent syphilis but had only been treated with a single dose of intramuscular penicillin followed by doxycycline for 2 weeks, due to a vasovagal reaction to penicillin.

The physical examination was unremarkable; renal and LFTs were normal. Platelet count was slightly elevated to 498/mm^3^, as well as C-reactive protein to 27 mg/L. His CD4 cell count was 556/mm^3^, and his HIV viral load was undetectable.

**Figure 1:**
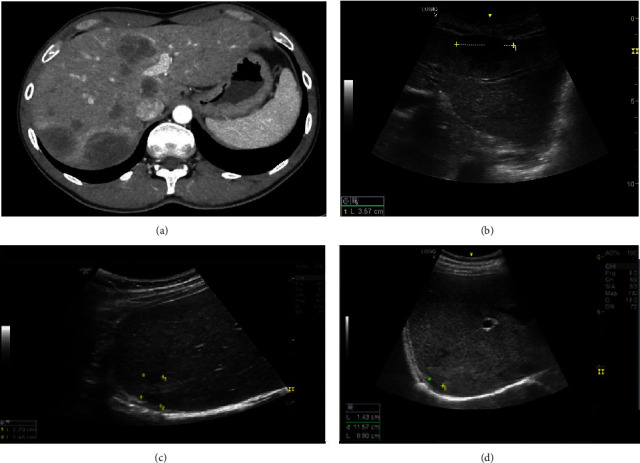
(a) A contrast-enhanced CT scan of the patient at the time of diagnosis. (b) A hypoechoic, round, well-defined lesion at the time of the first US scan. (c, d) US scan after treatment showing involution of the hypoechoic lesions shown at the first US.

Abdominal ultrasound (US) revealed multiple round hypoechoic hepatic lesions of varying sizes, the largest measuring 5 cm, along with periportal lymphadenopathy. Computed tomography (CT) of the abdomen confirmed the presence of multiple hepatic hypodense lesions, which showed peripheral enhancement following contrast administration (Figures 1(a) and 1(b) and Supporting Video 1).

Hepatitis B, C, and Entamoeba serologies as well as QuantiFERON-TB Gold were negative. Venereal Disease Research Laboratory (VDRL) and *Treponema pallidum* Hemagglutination Assay (TPHA) were both reactive (titers 1/16 and 1/5120, respectively). The patient underwent two percutaneous biopsies of liver lesions which demonstrated granulomatous inflammation with epithelioid cells, multinucleated giant cells, eosinophils, and coagulative necrosis. Bacterial culture, acid-fast bacilli smear microscopy, nuclear acid amplification test, and culture for mycobacteria on biopsies were negative.

Under suspicion of hepatic syphilis, the patient was subsequently treated with intravenous penicillin G 20,000,000 units per day for 2 weeks, using a dosing comparable to the ones used for cases of gummatous syphilis or neurosyphilis. At follow-up 1 month after treatment completion, abdominal pain, fever, and night sweats had resolved, and abdominal US showed a marked reduction in the number and size of his hepatic lesions. One year after therapy, the patient remained asymptomatic, and US demonstrated a reduction in the size of the hepatic lesions, with a change in the US appearance from hypoechoic to hyperechoic, suggesting the resolution of infection (Figures 1(c) and 1(d)).

## 3. Discussion

After secondary syphilis, the infection may enter a latent phase which can last years, with eventual progression to tertiary syphilis, characterized by the development of neurosyphilis, cardiovascular syphilis, or gummatous syphilis. Gummata are necrotizing granulomatous lesions, which are indolent and vary in size from small defects to large tumor-like masses. They are most commonly found in skin, mucocutaneous surfaces, and skeletal system but can develop in any organ including the liver [1, 8]. Hepatic gummata were once seen more frequently in visceral syphilis due to the wider use of antibiotics [8]. Gummatous hepatitis may clinically cause low-grade fever, epigastric pain, and tenderness like in our patient [9]. Of note, all LFTs in our patient were normal.

Although not well documented in the literature, case reports describe multiple circumscribed hypoechoic lesions on sonography. Our report describes multiple syphilitic hepatic gummata diagnosed by cross-sectional imaging. On CT, the centrally necrotic hepatic gummata tend to appear as multiple low-density lesions of varying sizes throughout both hepatic lobes, and they may infrequently demonstrate calcifications. Given mild peripheral enhancement after contrast administration, they can be virtually indistinguishable from abscesses caused by other organisms or from necrotic hepatic malignancies [8, 9].

Although both VDRL and TPHA were reactive in our patient—ultimately facilitating diagnosis—limitations of VDRL testing are important to recognize. Since nontreponemal tests like VDRL lose sensitivity in late syphilis, often yielding false-negative results, investigations with a treponemal test like the Fluorescent Treponemal Antibody Test (FTA-Abs) or TPHA are imperative [1] but can also return a false-positive result. Due to the absence of follow-up serologies carried out in the home country of the patient, we cannot know whether the patient had previously failed treatment or whether this was the consequence of reinfection. However, treatment failure is the most likely scenario since penicillins remain the only proven treatment for late latent syphilis [3]. A decision to treat with penicillin despite a previous vasovagal reaction to intramuscular penicillin was made as the history did not fit true allergy.

As observed in our patient, histological findings in gummatous hepatitis show areas of coagulation necrosis besides the granulomatous changes described above. These changes are suggestive but not fully specific of syphilis [9], and other infectious and noninfectious causes of necrotic granulomatous diseases such as tuberculosis, deep fungal infections, and sarcoidosis still need to be considered. In our case, Whartin–Starry staining of samples was not performed, and this could have increased diagnostic specificity [9].

Diagnosis of gummatous syphilis is therefore classified as “probable” in case of clinically compatible lesions and reactive serum treponemal test but only as “confirmed” when treponemes are identified in tissue sections (silver stain or immunohistochemistry) or DNA is detected in tissue by PCR.

While there are no specific treatment guidelines for syphilitic hepatitis or hepatic gummas, several reports describe favorable outcomes following treatment with intravenous penicillin; other guidelines recommend weekly intramuscular penicillin for the treatment of syphilitic hepatitis and gummatous syphilis [10].

In our case, the patient with the “probable” diagnosis of gummatous hepatitis was successfully treated with intravenous penicillin confirming the diagnosis.

## 4. Conclusion

Syphilis should be considered as a differential diagnosis in sexually active patients with abnormal hepatic function tests or liver parenchymal changes or lesions on US or cross-sectional imaging, especially since the imaging findings of hepatic involvement as described in the literature are nonspecific and may be easily initially attributed to any number of other disease entities. Although pyogenic and tubercular abscesses as well as neoplasms are more common causes of hepatic lesions in patients with HIV, syphilis should be consistently investigated as a potential etiology of hepatic inflammation.

## Data Availability

Data are available on reasonable request from the authors.

## Supporting Information

Supporting Information Supporting Video 1: Video showing the initial aspect of the gummatous lesions seen in the liver.
