# Supplementary material for: An Uncommon Manifestation of a Great Imitator: Gummatous Syphilis of the Liver in an HIV-Positive Patient
Source: Case Rep Infect Dis. 2024 Oct 21;2024:6571155. doi: 10.1155/2024/6571155 (PMC11519062; doi:10.1155/2024/6571155)
Supplement: Supporting Information — Supporting Video 1: Video showing the initial aspect of the gummatous lesions seen in the liver. [file 6571155.f1.docx]

<https://drive.google.com/file/d/1mT6Jv0bNMd-kv8_rvEE5QxCVqpnY4PJm/view?usp=sharing>
